# Reliability of Ultrasonographic Assessment of Depth of Invasion and Tumor Thickness in Intraoral Mucosa Lesions: A Preliminary Experience

**DOI:** 10.3390/jcm13092595

**Published:** 2024-04-28

**Authors:** Anna Russo, Vittorio Patanè, Luigia Fusco, Lorenzo Faggioni, Ciro Emiliano Boschetti, Mario Santagata, Emanuele Neri, Salvatore Cappabianca, Alfonso Reginelli

**Affiliations:** 1 Department of Precision Medicine, University of Campania Luigi Vanvitelli, 80131 Naples, Italy; 2 Department of Translational Research, Academic Radiology, University of Pisa, 56126 Pisa, Italy; lorenzo.faggioni@unipi.it (L.F.);

**Keywords:** high-frequency ultrasound, oral cancer, oral squamous cell carcinoma, ultrahigh-frequency ultrasound

## Abstract

**Introduction:** Despite the progress made in multidisciplinary care, there has been little improvement in the oncologic outcomes of oral cavity squamous cell carcinomas (OSCCs). In the latest edition of the TNM staging, “depth of invasion” (DOI) has recently been introduced as one of the criteria for determining the T stage, alongside other factors. DOI is widely recognized as an independent risk factor for nodal metastases and is a crucial consideration in the preoperative staging of OSCCs, along with measurements of tumor thickness (TT). While various diagnostic methods exist for assessing DOI, intraoral ultrasonography (IOUS) has gained popularity for its efficacy in evaluating OSCCs. **Methods:** This study sought to evaluate the diagnostic accuracy and reliability of ultrahigh-frequency ultrasound (UHFUS) in assessing oral cavity lesions compared to histopathological analysis. **Results:** The results revealed strong reliability in ultrasonographic measurements (ICC TT: 0.94; ICC DOI: 0.97) and distinct ultrasonographic features specific to different oral pathologies. This highlights the potential of UHFUS as a non-invasive imaging tool for precise diagnostic evaluations. **Conclusions:** Despite limitations such as a small sample size and focus on specific lesions, these promising results suggest that UHFUS could significantly enhance oral lesion diagnostics. Further research involving larger cohorts is necessary to validate and build upon these initial findings.

## 1. Introduction

Oral squamous cell carcinomas (SCCs) are recognized as the most prevalent neoplasms affecting the head and neck region, primarily originating in the oral tongue [1]. Oral cavity squamous cell carcinomas (OSCCs) represent a substantial global burden of cancer, with over fifty thousand new cases reported annually in the United States alone, accompanied by an estimated cancer-specific mortality rate of around 30% [2]. Despite advances in surgical methodologies and comprehensive care strategies over the past twenty years, the oncological outcomes for OSCC patients have shown limited enhancement [3]. While early-stage overall survival and disease-specific survival rates are acceptable, the risk of lymphatic spread remains notably high and serves as a critical prognostic indicator [4]. Moreover, the incidence of occult nodal metastasis in clinically negative necks varies significantly, with a substantial increase in mortality rates associated with the presence of occult nodal metastases [5].

As of now, the gold standard for diagnosis is a biopsy for histopathological examination; nonetheless, emerging imaging techniques are being evaluated to streamline and enhance the diagnostic process [6,7].

The assessment of depth of invasion (DOI) was integrated into the 8th edition of the American Joint Committee on Cancer–Union for International Cancer Control (AJCC-UICC) TNM staging system for stages 1, 2, and 3 [8]. DOI emerged as a more dependable prognostic indicator compared to tumor thickness (TT), as it can differentiate between superficial and deeply invasive tumors [9]. Additionally, DOI may serve as a predictor for the presence of regional nodal metastases. Beyond its prognostic value, DOI estimation can enhance surgical planning by providing insights into the depth of tumor invasion, potentially guiding decisions regarding the extent of resection and the need for prophylactic neck dissection in cases where the DOI is equal to or greater than 4 mm [9]. In the realm of preoperative assessment, the identification of aggressive features in OSCCs would allow surgeons to anticipate the tumor’s biological behavior, thus facilitating tailored treatment strategies, especially for early-stage patients [10]. Radiological techniques such as computed tomography (CT) and magnetic resonance imaging (MRI) are commonly utilized to assess the extension of OSCCs, with MRI being particularly preferred even for smaller lesions [11,12].

Ultrasonography (US) has become a versatile tool in various medical specialties, including gynecology, gastroenterology, cardiology, and angiology [13,14]. Within the field of head and neck imaging, it has proven valuable for detecting abnormalities and malignancies in salivary glands [6,7]. Moreover, its applications extend to studying periodontal tissues and characterizing lesions of the oral mucosa for clinical and presurgical purposes [1,13,15].

UHFUS shows potential across various medical domains owing to its capability of delivering real-time imaging, eliminating the risk of ionizing radiation exposure, and ensuring consistency in lesion follow-up assessments. As emphasized by the findings presented in this Special Issue, current research corroborates the growing adoption of this method in diagnosing, prognosticating, treating, and monitoring numerous diseases and conditions. Shintani et al. were pioneers in deciphering the sonographic characteristics of healthy oral mucosa. They observed a homogeneous ultrasonographic pattern in the healthy tongue and buccal mucosa. The normal buccal mucosa exhibited a homogeneous pattern with a hyperechoic appearance attributed to the thick cortical bone of the mandible. Additionally, regular patterns of the mouth floor were characterized by acoustical shadowing due to the sublingual glands [16]. However, their studies utilized equipment with relatively low frequency emission, only 7.5 MHz. Advancements in technology have enabled recent ex vivo and in vivo studies to leverage high-frequency US, which can discern tissue layers based on image contrast. This contrast arises from acoustic backscatter, which is directly proportional to density changes at tissue boundaries. Consequently, less dense fat tissue layers appear hypoechoic, while denser tissue layers such as muscle and connective tissue appear hyperechoic on images [17]. This enhancement in technology offers greater detail and resolution in imaging, aiding in the precise characterization of various oral mucosal lesions and structures. However, ultrasound (US), historically limited to evaluating the neck, thyroid gland, or salivary glands, has garnered attention in OSCC evaluation, particularly with the introduction of advancements in ultrasonographic technologies like high- and ultrahigh-frequency ultrasound (HFUS and UHFUS) [18]. When it comes to skin tumors, UHFUS can aid in diagnosing and surgically treating various neoplastic conditions. Laverde-Saad [19] noted that UHFUS accurately measured the depth of basal cell carcinomas, particularly when lesions exceeded 1 mm. Specifically, the technique allowed for evaluating tumor depth and margins, as well as distinguishing between aggressive and non-aggressive subtypes. Regarding melanoma skin lesions, a systematic review by Dinnes and colleagues [20] found sensitivities of at least 83% and combined the assessment of three qualitative features: hypoechogenicity, homogeneity, and well-defined margins. Moreover, UHFUS has been shown to correlate the Breslow scale with ultrasonographic thickness [18,19]. The strong correlation between UHFUS and histology could enhance the surgical treatment of these lesions, improving the ability to achieve clear resection margins—a critical factor in prognosis and preventing recurrence. Nonetheless, it is worth noting that UHFUS, when used in conjunction with dermoscopy, can also be advantageous in the diagnostic evaluation of non-melanoma skin tumors [21,22,23].

Recent studies have highlighted the potential of high-frequency intraoral ultrasonography (IOUS) in providing precise preoperative evaluation of DOI and TT due to its excellent spatial resolution [8,22,24]. Ultrahigh-frequency ultrasound (UHFUS), although initially utilized in dermatology and angiology, holds promise in oral lesion diagnostics by enabling accurate imaging of superficial epithelial layers [25]. This study aims to assess the diagnostic accuracy in terms of reliability of HFUS in evaluating tumor thickness (TT) and depth of invasion (DOI) in oral cavity lesions compared to histopathological counterpart assessment.

## 2. Methods

The study’s protocol was approved by the local ethics committee at the University Hospital of Campania “L. Vanvitelli” and AORN “Ospedale dei Colli”, Naples, Prot. N. 13985/i/2022. Informed consent was obtained from all subjects involved in the study. The study adhered to the principles outlined in the Declaration of Helsinki regarding experimentation involving human subjects.

### 2.1. Patient Criteria

Patients referred to the Unit of Maxillo-Facial and Oral Surgery at the University Hospital “Luigi Vanvitelli” in Naples, Italy, were included in this study and competitively enrolled in the period between May 2022 and October 2023.

To be eligible, participants needed to meet the following criteria: adult aged 18 or older, clinical diagnosis of an oral soft tissue lesion existing for more than 15 days, suitability for surgical biopsy, good health status or well-controlled chronic conditions with medication, and willingness to participate in the study. Exclusion criteria comprised severe comorbidities resulting in a life expectancy of less than 1 year, pregnancy, acute or chronic conditions hindering study participation, or refusal to provide informed consent.

### 2.2. UHFUS Scan Protocol

Oral soft tissue lesions were examined utilizing a 70 MHz “hockey stick” probe, which had been available at the institute since 2016. Each participant underwent an intraoral UHFUS examination using the Vevo^®^ MD device from VisualSonics in Toronto, ON, Canada. Intraoral ultrasounds were conducted prior to biopsy by an experienced radiologist with over 10 years of experience in the field. The radiologist utilized a high-frequency “hockey stick” probe (22–28 MHz) covered with a sterile latex sheath. A small amount of ultrasound gel was placed inside the cover to create a distance between the probe and the mucosa, facilitating clear visualization of exophytic or ulcerated lesions. Gentle pressure was applied to minimize compression distortion of the lesion’s thickness and morphology. B-mode and C-mode acquisitions were conducted for each lesion using standardized presets, maintaining constant parameters such as gain, time gain compensation, dynamic range, mechanical index, and thermal index. The scan depth and focus position were adjusted as needed to optimize the scan quality. The ultrahigh-frequency ultrasound (UHFUS) examination was performed by an experienced radiologist with over 10 years of expertise. DOI was measured according to the NCCN guidelines, from the mucosal surface to the deepest point of infiltration along a perpendicular line, excluding exophytic parts and including ulcerated portions of the lesions. Imaging features were retrospectively analyzed on static anonymized images utilizing the institutional Picture Archival and Communication System (PACS). TT, measured in millimeters, represented the distance between the lesion’s surface and the deepest margin in the tissue. DOI, also measured in millimeters, indicated the distance between the normal mucosal surface and the deepest margin of the lesion in the tissue.

### 2.3. Histopathological Assessment

Pathological assessment was conducted by a pathologist with more than 10 years of experience in the field.

Pathological tumor thickness (pTT) was assessed from the tumor surface to its deepest point of invasion. In cases of ulcerated tumors, an alternative definition of pTT was utilized, measuring from the ulcer bed to the deepest point of invasion, as proposed by the AJCC 7th edition. Pathological depth of invasion (pDOI) was measured from an imaginary line connecting the basement membrane of normal squamous mucosa on both sides of the tumors perpendicular to the deepest point of invasion.

### 2.4. Data Collection

Two specific parameters, TT and DOI, were recorded for all patients who underwent UHFUS. TT is measured as the largest dimension along the predominant axis of the lesion (length or width) and is expressed in millimeters. The DOI is determined as the extension measured in millimeters between the free surface of the tumor and the deep margin.

The gathering and utilization of data for this study received approval from the Institutional Ethics Review Board of our institution’s ethics committee.

### 2.5. Statistical Analysis

These parameters were analyzed using the interclass coefficient of correlation (ICC) to evaluate the concordance between ultrasonographic and in vivo histopathological measurements.

## 3. Results

### 3.1. Sample Characteristics

**Figure 1:**
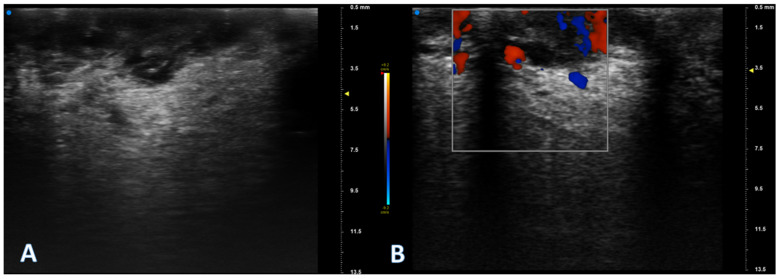
The high-frequency ultrasound (**A**) reveals an irregular localized thickened area within the oral mucosa presenting as a hypoechoic lesion. The lesion appears to have a slightly irregular surface with the normal free margin presenting as focally interrupted and is confined to the superficial layers of the mucosa. No infiltration into deeper tissues is noted, with globally deeper regular margins. Surrounding tissues show normal echogenicity with no evident signs of metastasis or structural abnormalities. In addition, Doppler signal analysis (**B**) reveals an increase in vascularity compared to normal tissues. Vascularity is notably peripheral, showing an increase in both arterial and venous components.

**Figure 2:**
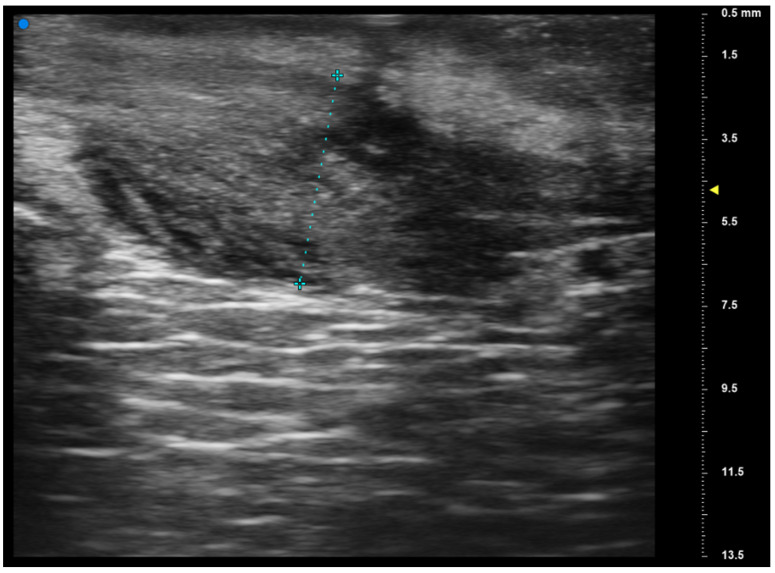
The high-frequency ultrasound reveals an irregular, hypoechoic lesion within the oral mucosa, which is consistent with oral lichen planus. The lesion exhibits somewhat irregular borders mainly in the upper margins and is predominantly localized in the superficial layers of the mucosa. There is no clear evidence of invasion into deeper tissues. Surrounding tissues display normal echogenicity without apparent signs of metastasis or structural irregularities. Imaging findings are not univocally clear, and at the end of the HFUS examination it was still not clear if the lesion was a squamous carcinoma or a lichen planus. Final histopathological examination determined that the lesion was a lichen planus.

During the research period extending from May 2022 to November 2023, a cohort of 44 individuals was examined. Their ages ranged from 58 to 86 years, with a median age around 70 years, and there was a slight male predominance (male/female ratio of 6:5). Among the participants, twenty-four were identified as smokers, with an average consumption of 18.3 cigarettes per day. Twelve patients had concurrent health conditions, which included two cases of previous breast cancer and one with liver damage related to HCV. Within this group, twenty-six individuals were diagnosed with squamous cell carcinoma of the oral cavity (Figure 1), four had angioleiomyoma, six were diagnosed with hyperkeratosis following biopsy, and four exhibited lichen planus (Figure 2). Twenty patients reported either no pain or mild pain (rated < 4 on the Visual Analogue Scale—VAS), while twenty-four patients experienced more intense pain levels (>6 on the VAS). The average VAS score for all patients was 5.1. Notably, patients with endophytic lesions had significantly lower VAS scores (mean: 3.75 ± 3.10, *p* < 0.05) compared to those with exophytic lesions (mean: 6.33 ± 3.09). The remaining twenty patients did not report any pain.

**Figure 3:**
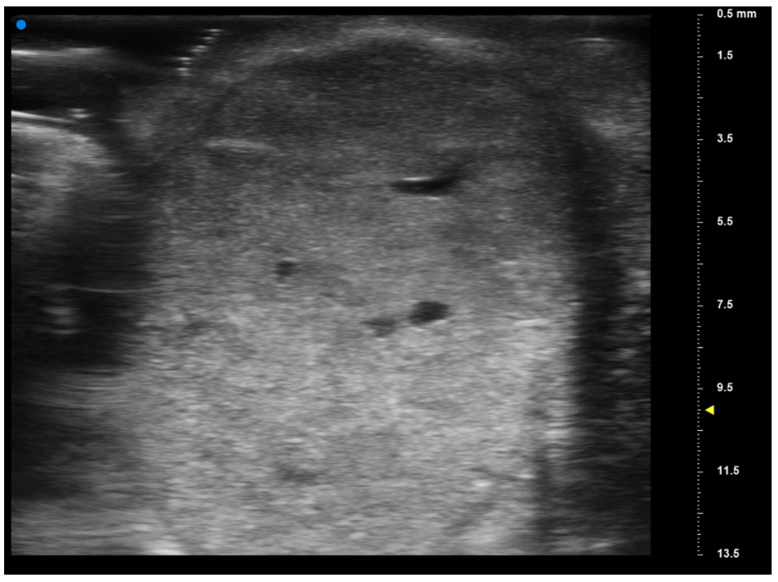
The high-frequency ultrasound depicts a well-defined round-shaped inhomogeneous hyperechoic lesion within the oral mucosa consistent with a mucoepidermoid carcinoma. The lesion exhibits regular borders and is confined to the deeper layers of the mucosa. There are heterogeneous areas within the lesion, reflecting mixed echogenicity, suggestive of varying tissue compositions. Surrounding tissues do not show any apparent signs of invasion.

Four patients received a final diagnosis of mucoepidermoid carcinoma (Figure 3).

### 3.2. Interclass Coefficient Correlation

**Figure 4:**
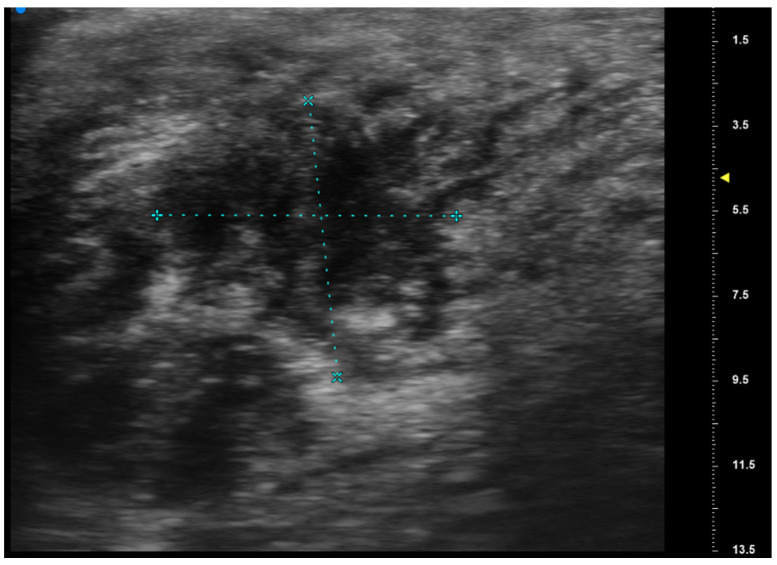
The high-frequency ultrasound depicts an ill-defined hypoechoic lesion located in the oral mucosa. The lesion appears irregular in its contours with deep infiltration into the underlying tissues with dendritic margins. There are coexisting variations in reflectivity and an alteration in the surrounding tissue structure. There are no evident signs of regional metastasis in adjacent structures.

After computing the interclass correlation coefficient, the statistical analysis demonstrated excellent reliability for the test of each individual parameter (ICC TT: 0.94; ICC DOI: 0.97). The initial results provided intriguing insights into the ultrasonographic features of different oral pathologies. Particularly, infiltrating squamous carcinomas displayed distinct hypoechoic patterns with irregular, stellate borders and increased vascularity surrounding the mucosal and submucosal areas (Figure 4).

**Figure 5:**
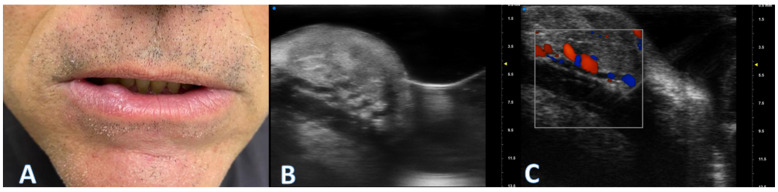
(**A**) Patient presenting with a round-shaped mildly consistent subepithelial formation. Overlying skin shows no dyschromia or any other significant alteration. (**B**) The high-frequency ultrasound reveals a well-defined, hyperechoic mass within the oral labial mucosa consistent with an angioleiomyoma. The lesion displays regular borders and appears to be encapsulated and localized within the superficial layers of the mucosa. Vascular structures are visible within the mass as areas of hypoechoic focal inhomogeneity, demonstrating hypervascularity upon Doppler imaging (**C**). Surrounding tissues exhibit normal echogenicity without signs of infiltration or structural alterations.

In contrast, conditions like candidiasis and fibrous dysplasia exhibited noticeable ultrasonographic characteristics. Candidiasis often presented with heterogeneous submucosal involvement, while fibrous dysplasia typically appeared as isoechoic exophytic lesions with clearly defined margins (see Figure 5).

## 4. Discussion

In Europe, approximately 66,650 new cases of oral cancer are diagnosed annually [4,26]. Despite the oral cavity being easily accessible for self-examination, diagnoses often occur at advanced stages [27]. Therefore, thorough investigation of all upper aero-digestive tracts is recommended, as risk factors for oral cavity tumors align with those of other areas such as the oropharynx, larynx, and hypopharynx [28]. Palpation along with fibroscopy remains crucial for identifying primary lesions and detecting suspicious swellings in the neck. Despite advancements in treatment, survival rates for patients with oral squamous cell carcinomas (OSCCs) have not notably improved over time [29]. Nodal metastases and recurrences are not uncommon, even in earlier stages of the disease. Magnetic resonance imaging is considered the preferred method for staging oral squamous cell carcinomas (OSCCs) due to its outstanding soft tissue resolution. Computed tomography (CT) can offer additional insights into cortical bone erosions, complementing MRI findings. Ultrasound, on the other hand, is a valuable non-invasive tool for evaluating various body regions, including the head and neck, where it serves as the primary method for examining neck swelling. It provides detailed anatomical information, particularly regarding nodal stations in the neck. Our initial observations suggest that preoperative assessment of tumor thickness (TT) and depth of invasion (DOI) parameters using ultrahigh-frequency ultrasound (UHFUS) correlates significantly with histological findings [23].

The ultrasound analysis of neoplasms spans various anatomical areas, including the skin, thyroid, salivary glands, breasts, lungs, and pancreas [30,31,32,33,34,35]. Each area presents unique anatomical features that influence ultrasound appearance, necessitating distinct evaluation parameters. Several biases must be considered when comparing ultrasound studies across different anatomical districts and neoplasms:

(a) Qualitative differences between probes: variations in probe performance and frequency emission can impact image quality and resolution, influencing the interpretation of ultrasound findings.
(b) Intrinsic differences in anatomical areas: variations in tissue composition, thickness, and organization contribute to differences in ultrasound appearance between anatomical districts.
(c) Intrinsic differences in tumor histology: variances in tumor histology across different neoplasms can lead to diverse ultrasound characteristics, complicating comparative analysis.

Given these limitations, a natural comparison can be drawn between oral squamous cell carcinomas (OSCCs) and skin neoplasms, particularly squamous cell carcinomas (SCCs) and squamous cell basal cell carcinomas (BCCs). The skin shares similarities with the oral cavity in terms of epithelial coating and layered structure, facilitating a more straightforward comparison.

The existing literature predominantly focuses on ultrasound assessment of melanoma in the skin, particularly utilizing ultrasound Breslow thickness for preoperative evaluation and postoperative follow-up [20]. In contrast, SCCs and BCCs of the skin exhibit distinct ultrasound characteristics [36]. BCCs typically appear as well-defined, oval, or slightly irregular hypoechoic lesions with hyperechoic spots representing neoplasm nests. On the other hand, SCCs present as heterogeneously hypoechoic lesions with irregular borders, lacking hyperechoic spots and involving deeper layers [37].

In OSCCs, ultrasound findings reveal specific characteristics related to grading and invasiveness detected by pathological analysis. OSCC lesions are typically well-defined and distinguishable from surrounding tissues, with variations in invasiveness impacting the delineation of deep layers. Invasive carcinomas often exhibit a relatively well-defined hypoechoic lesion pattern, contrasting with the homogeneous echogenicity of surrounding tissues [38]. However, certain features, such as hyperechoic images due to air interpositioning in ulcerated areas, were not observed in the current study.

In summary, when comparing ultrasound findings across different anatomical districts and neoplasms, it is essential to account for inherent biases and variations in ultrasound characteristics influenced by tissue composition, probe performance, and tumor histology. Comparing OSCCs with skin neoplasms such as SCCs and BCCs offers valuable insights due to similarities in epithelial structure and layered organization.

UHFUS tends to slightly overestimate DOI measurements, while TT is underestimated by approximately 0.1 mm, likely due to enhanced visualization of tumor margins with higher frequencies. A possible explanation for this particular aspect may reside in the HFUS technology itself. In particular, when using higher frequencies we have a less deep plane of penetration of the sound waves, thus causing fewer echoes coming back from the several tissues they meet along the way. This may explain the reduced definition in the deepest layer of tissue which causes the smooth underestimation of some parameters (e.g., DOI). However, it is important to acknowledge that histological TT measurements were marginally higher than those obtained via UHFUS, although this difference was not statistically significant. This variance could be attributed to minimal pressure during UHFUS scanning, resulting in slightly reduced TT dimensions compared to histological measurements.

Intraoral ultrasound (IOUS) has long been acknowledged for its prognostic significance in the management of oral cancer [22]. Historically, TT was commonly utilized before the introduction of DOI for assessing tumor depth. Although both TT and DOI are linked to the risk of nodal metastases, DOI is preferred due to its higher reliability. However, combining evaluations of TT and DOI can enhance staging and prognosis, particularly for smaller tumors where occult metastases are a concern [39,40].

Numerous studies have demonstrated strong correlations between ultrasound (US) TT measurements and histology, especially for tumors larger than 10.0 mm [38,41,42]. US assessments provide predictive insights into nodal metastases and can influence treatment decisions. The relationship between DOI and histology suggests a role in evaluating tumor aggressiveness and guiding treatment strategies [11,43].

While the application of intraoral ultrasound (IOUS) may seem unconventional, interest in its utilization for OSCC staging is rapidly increasing. Several studies have compared ultrasonographic DOI with other modalities such as MRI and CT, yielding promising results despite some notable limitations. However, it is essential to consider this study as a preliminary endeavor, necessitating further investigations with larger sample sizes to validate and confirm the findings.

Future research should focus on comparing ultrahigh-frequency ultrasound (UHFUS) with conventional US and correlating echogenicity and vascularization with histological and clinical parameters. Nonetheless, our initial findings highlight the potential of UHFUS in the evaluation and surgical management of OSCC.

The use of high-frequency intraoral ultrasonographic examination of the oral mucosa remains relatively uncommon in many healthcare centers. However, both our study and the existing literature have demonstrated its promising potential [44,45,46,47]. Our research aimed to evaluate the diagnostic accuracy of high-frequency ultrasound (HFUS) in assessing TT and DOI in oral cavity lesions. Analysis of ultrasonographic measurements, including TT and DOI, revealed high reliability, as indicated by excellent interclass correlation coefficients (ICCs) for both TT (ICC: 0.94) and DOI (ICC: 0.97). We observed distinct ultrasonographic characteristics among different oral pathologies, with squamous cell carcinomas displaying unique features such as hypoechoic structures with irregular borders and increased vascularity. These initial findings highlight the potential of UHFUS as a non-invasive imaging tool, offering valuable insights into various oral pathologies. However, our study had limitations, such as a small sample size and a focus on specific lesions. Further research involving larger cohorts and diverse oral pathologies is necessary to validate and build upon our preliminary findings. Nevertheless, the encouraging results suggest that UHFUS could significantly impact the diagnostic approach to oral cavity lesions, potentially enhancing early detection and optimizing treatment strategies.

## 5. Conclusions

In conclusion, despite its current limitations in visualizing specific lesions on the posterior palate, intraoral ultrahigh-frequency ultrasound (UHFUS) demonstrates promising potential for assessing oral lesions, particularly in cases of oral squamous cell carcinomas (OSCCs). The observed consistency between UHFUS imaging and pathological anatomy, albeit in a limited sample size, emphasizes its relevance as a valuable adjunctive tool in diagnostic and surgical protocols. This study advocates for the integration of UHFUS into the diagnostic armamentarium for assessing oral lesions, potentially enhancing the precision of diagnosis and subsequent therapeutic strategies in managing OSCCs. Our findings provide an initial step toward addressing the demand for a dependable, non-invasive, and economical method to predict adverse histological features preoperatively. Moving forward, head and neck cancer specialists will need the capability and suitable tools to tailor the extent of resection according to radiological parameters that can act as markers for underlying adverse pathological traits.

## Data Availability

Data of the reported results is available from the corresponding author upon request.
